# Genome Sequence of “*Candidatus* Carsonella ruddii” Strain BC, a Nutritional Endosymbiont of *Bactericera cockerelli*

**DOI:** 10.1128/genomeA.00236-17

**Published:** 2017-04-27

**Authors:** Alex B. Riley, Dohyup Kim, Allison K. Hansen

**Affiliations:** Department of Entomology, University of Illinois at Urbana-Champaign, Urbana, Illinois, USA

## Abstract

Here, we report the genome of “*Candidatus* Carsonella ruddii” strain BC, a nutritional endosymbiont of the tomato psyllid *Bactericera cockerelli*. The 173,802-bp genome contains 198 protein-coding genes, with a G+C content of 14.8%.

## GENOME ANNOUNCEMENT

Insects in the family Psyllidae are vectors for a number of plant pathogens, making them agriculturally and economically important (1–3). Because they feed on nutrient poor phloem sap, psyllids depend on their obligate bacterial endosymbiont “*Candidatus* Carsonella ruddii” as a source of essential amino acids (4). Each species of psyllid harbors a specific strain of “*Ca.* Carsonella ruddii,” which is maternally transmitted. The genomes of “*Ca.* Carsonella ruddii” strains are highly reduced (158 kb to 174 kb) (4–6), making them biologically interesting organisms. Here, we present the sequenced genome of “*Ca.* Carsonella ruddii” BC, which is associated with the tomato psyllid *Bactericera cockerelli*.

Bacteriocytes, specialized insect cells that harbor “*Ca*. Carsonella,” from 10 tomato psyllids were dissected and pooled, and DNA was extracted. The extracted DNA was prepared using the Nextera DNA library preparation kit (Illumina, San Diego, CA) and sequenced within a 1/4 of a lane on an Illumina HiSeq 2500 (Illumina) using TruSeq SBS sequencing chemistry (Illumina). Fastq files were generated with the software Casava 1.8.2 (Illumina). The initial assembly and quality trimming were performed using a customized A5ud pipeline, which was modified from the A5 genome assembly pipeline (7). Further assembly was done using GapFiller (8) and SEQuel version 1.0.2 (9). Due to the high content of insect DNA in the sequencing reads, the contigs and scaffolds were filtered by G+C content and contig lengths. In order to complete the genome assembly, filtered reads and contigs were reassembled into gapped regions using Mimicking Intelligent Read Assembly (MIRA) version 4.0.2 (10). The remaining gaps were closed using Sanger sequencing of PCR products. The assembled genome was annotated using both the Rapid Annotation using Subsystem Technology (RAST) (11) and the Bacterial Annotation System (BASys) servers (12).

The complete circular genome of “*Ca.* Carsonella ruddii” BC is 173,802 bp long, with 198 protein-coding genes and a G+C content of 14.8%. Genes involved in essential amino acid biosynthesis that are present and absent in all other “*Ca.* Carsonella” strains sequenced to date have the same pattern of presence and absence in “*Ca.* Carsonella ruddii” BC (4, 5, 13). One exception, however, is that *pheA*, a gene conserved in all other sequenced “*Ca.* Carsonella” genomes to date and that is involved in phenylalanine biosynthesis, is missing from “*Ca.* Carsonella ruddii” BC’s genome. Potentially, a homolog of *pheA* that is microbe in origin has been horizontally transferred to *Bactericera cockerelli*’s genome to complement “*Ca.* Carsonella ruddii” BC’s essential amino acid pathways, analogous to mealybug, whitefly, and psyllid obligate symbiont symbioses (13–15). In sum, our genome sequence supports the role of “*Ca.* Carsonella ruddii” BC for the biosynthesis of essential amino acids for its insect host, *Bactericera cockerelli*.

### Accession number(s).

The complete genome sequence of “*Ca.* Carsonella ruddii” BC and its annotation are deposited at GenBank, NCBI, under accession number CP019943.
